# Identification of chemical constituents of *Zanthoxylum heitzii* stem bark and their insecticidal activity against the malaria mosquito *Anopheles gambiae*

**DOI:** 10.1186/s13071-015-1113-x

**Published:** 2015-10-01

**Authors:** Nastaran Moussavi, Karl Egil Malterud, Bertin Mikolo, Dag Dawes, Fabrice Chandre, Vincent Corbel, Daniel Massamba, Hans J. Overgaard, Helle Wangensteen

**Affiliations:** School of Pharmacy, Department of Pharmaceutical Chemistry, Section Pharmacognosy, University of Oslo, P.O. Box 1068 Blindern, 0316 Oslo, Norway; National Polytechnic High School, Marien Ngouabi University, BP 69, Brazzaville, Republic of Congo; Institut de Recherche pour le Développement (IRD), Maladies Infectieuses et Vecteurs, Ecologie, Génétique, Evolution et Contrôle (IRD 224-CNRS 5290 UM1-UM2), Montpellier, Cedex 5 France; Department of Mathematical Sciences and Technology, Norwegian University of Life Sciences, P.O. Box 5003, Ås, Norway; Department of Entomology, Kasetsart University, Bangkok, Thailand

**Keywords:** *Zanthoxylum heitzii*, *Anopheles gambiae*, Pellitorine, Malaria, Insecticide

## Abstract

**Background:**

*Zanthoxylum heitzii* bark extracts have insecticidal properties and have been reported to be used against malaria in Western Africa. Previously, it has been shown that a hexane extract of the bark is toxic to adult females of the mosquito *Anopheles gambiae*, a malaria vector. As part of our project on the control of malaria vectors using plant extracts, the phytochemistry of *Z. heitzii* bark hexane extract has been investigated with the aim to identify the major components with adulticidal and larvicidal effects on *An. gambiae*.

**Methods:**

*Z. heitzii* stem bark was extracted with hexane, and the extract was fractionated to isolate major components from the bark, identified by NMR spectroscopy. Isolated compounds were tested for toxicity towards adult female *An. gambiae* mosquitoes and for larvicidal effects towards *An. gambiae*.

**Results:**

The alkaloid dihydronitidine, the sesquiterpenoid caryophyllene oxide, the amide pellitorine and the lignan sesamin were identified as the major constituents in *Z. heitzii* bark. Pellitorine was toxic to both adult insects (LD_50_ 50 ng/mg insect) and larvae (LD_50_ 13 μg/ml). None of the other compounds were toxic to adults, but caryophyllene oxide and sesamin exhibited moderate larvicidal effects (LD_50_ > 150 μg/ml). A mixture of the four compounds in the same ratio as in the hexane extract showed higher toxicity (LD_50_ 34 ng/mg insect) towards adult insects than the pure compounds.

**Conclusion:**

The toxicity of *Z. heitzii* bark hexane extract to *An. gambiae* is mostly due to pellitorine, although interactions between pellitorine and other, inactive constituents may enhance the activity of the extract.

## Background

Malaria infection is estimated to kill more than 600 000 people every year, the majority of which are children below five years old [1]. Insecticides to kill or repellents to deter mosquitoes from biting are the mainstay of malaria vector control. However, mosquito resistance to commonly used insecticides (e.g. pyrethroids) is a major challenge [2]. There is therefore an urgent need for new and alternative insecticides. The tree *Zanthoxylum heitzii* (Aubrév. & Pellegr.) P.G. Waterman, syn. *Fagara heitzii* Aubrév. & Pellegr., Rutaceae, is a West African species found in forests from Congo to Cameroon [3]. Some of its local names are olon [3] and bouboulou [4]. This tree is used for timber, but has also a considerable ethnopharmacological use. The diseases for which it is used include jaundice [5], toothache [6], gonorrhoea [7], rheumatic ailments and stiff joints, impotence [3, 7] and malaria [3]. It has also been used as a fish poison [3]. Chemically and pharmacologically, this plant has only been subjected to a limited amount of research. This species has been shown to contain alkaloids, phenols, saponins, mucilage [8] and terpenoids [9]. More specifically, the alkaloids arnottianamide, fagaramide, iso-γ-fagarine, iso-γ-skimmianine, skimmianine and nitidine have been reported from the bark [10–12], flindersine [13] from the wood, and 6-methylnitidine [12] and iso-γ-skimmianine [10] from the roots. Two novel amides, heitziamide A and B, and two novel aromatic fatty acid esters, heitziethanoid A and B, were reported from the bark, as well as methyl esters of long-chain fatty acids [10]. The bark contains a variety of lignans [10, 11], and sterols and triterpenes have also been isolated from the bark or roots [10, 12]. *Z. heitzii* extracts have been shown to be active against Gram-positive bacteria [14], filarial worms [9], and two different cancer cell lines [14]. Antioxidant effects and activity against sickle cell anemia *in vitro* are reported [8], as well as immunorestorative properties of an aqueous bark extract in clinical studies [15]. The bark extract was also toxic towards agricultural weevil pests and the cockroach *Periplaneta americana* L. [4]. The effect of *Z. heitzii* extracts on adult females of the mosquito *Anopheles gambiae* Giles, a major vector of malaria, has recently been investigated by us [16]. After extracting diverse plant parts from *Z. heitzii* with solvents of different polarities, the hexane stem bark extract was found to be the most active against *An. gambiae*. The aim of this study was to identify the major components from the hexane stem bark extract with adulticidal and larvicidal effects on *An. gambiae*.

## Methods

### Plant material

Stem bark of *Zanthoxylum heitzii* was taken from a tree in Douakani, Republic of Congo, in November 2011. The tree was identified by one of the authors (B. Mikolo). A voucher sample of the bark is kept in the Section of Pharmacognosy, School of Pharmacy, University of Oslo (registry number ZH-B-111202).

### Preparation of extract

**Fig. 1:**
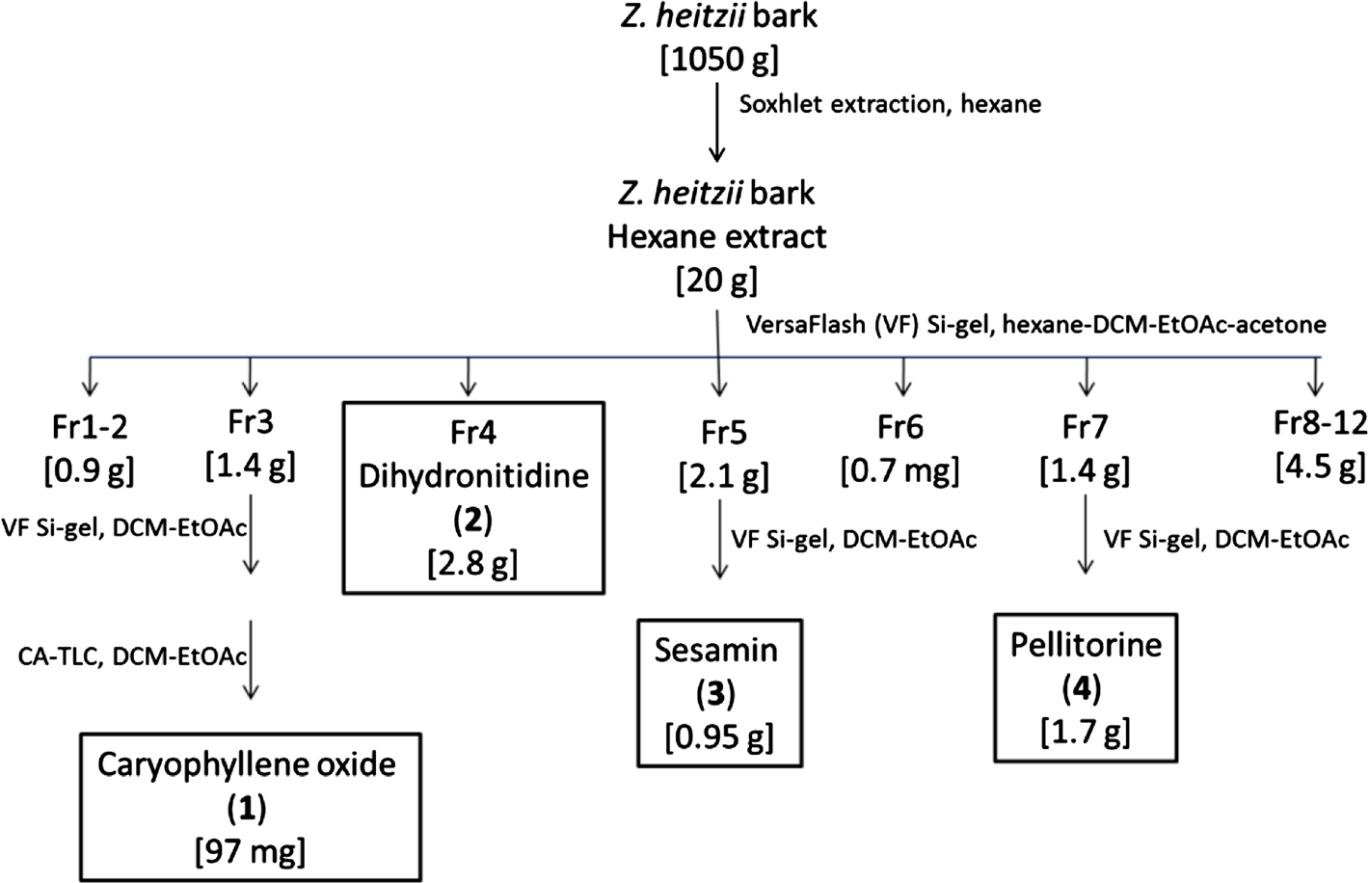
Flow scheme for extraction and isolation of compounds from *Zanthoxylum heitzii* bark. Abbreviations: VF: VersaFlash chromatography; CA-TLC: centrifugally accelerated thin layer chromatography; DCM: dichloromethane; EtOAc: ethyl acetate

The bark was air-dried and milled in a knife mill (4 mm sieve). Of the powdered bark, aliquots of ca 300 g were extracted with 3 liter portions of hexane in a Soxhlet extractor for 10 h. After cooling to room temperature, the solvent was removed on a rotary evaporator, and the dry extracts weighed. Average yield of extract was ca 1.9 % (w/w). A scheme of the extraction and fractionation processes is shown in Fig. 1.

### General experimental procedures

Column chromatographic separation was done on pre-packed Versapak normal phase Si gel columns (VersaFlash system; Supelco, Bellefonte, PA, USA) and preparative centrifugally accelerated thin-layer chromatography (CA-TLC) on a Chromatotron model 7924 T (Harrison Research, Palo Alto, CA, USA), using 1 or 2 mm layers of Si gel 60PF_254_ containing gypsum (Merck, Darmstadt, Germany). Analytical TLC was carried out on 0.2 mm Si gel 60 F_254_ plates (Merck). Spots were visualized by irradiation with short-wave (254 nm) and long-wave (366 nm) UV rays (UVGL-58 instrument, Ultra-Violet Products, Upland, CA, USA) and by spraying with a 1 % solution of Ce(SO_4_)_2_ in 10 % aqueous H_2_SO_4_ followed by heating to 105 ^o^C for 5 min. One- and two-dimensional NMR spectra were recorded in CDCl_3_ solution on a Bruker DPX300 instrument or a Bruker AVII400 instrument (Bruker, Rheinstetten, Germany) at 300 MHz for ^1^H/75 MHz for ^13^C and 400 MHz for ^1^H/100 MHz for ^13^C, respectively. HPLC analysis was performed on a LaChrom Elite HPLC system (Hitachi, Tokyo, Japan) equipped with an L-2455 diode array detector. A Chromolith Performance RP18e 100 x 4.6 mm column (Merck) was used for separation. Elution was performed using a gradient of mobile phase A (water) and mobile phase B (acetonitrile) with the following time schedule: 20 % B, 0–1 min; 20–95 % B, 1–15 min; 95 % B, 15–16 min. The concentration of injected samples was 0.5 mg/mL, injection volume was 10 μL and flow rate was 3.0 mL/min. The absorbance was recorded at 237 nm, and separation took place at 25 °C. All chemicals and solvents were of the highest quality available.

### Fractionation of the crude extract

Ca 20 g of the dried hexane extract of *Z. heitzii* stem bark was dissolved in 100 mL dichloromethane (DCM), filtered, and applied to a Versapak Si gel column (110 x 300 mm) conditioned with hexane-DCM, 1:1. The column was eluted with a hexane—DCM—ethyl acetate (EtOAc)—acetone gradient, and 37 fractions of 0.15 – 0.5 L were collected and combined into major fractions Fr1-Fr12 as indicated by analytical TLC (mobile phase DCM or DCM-EtOAc, 1:1). All combined fractions were subjected to ^1^H NMR spectroscopy.

### Purification and isolation of compounds

Fraction Fr3 (ca 1.4 g) from the crude extract was rechromatographed on a Versapak Si gel column (40 x 150 mm) with a DCM—EtOAc gradient, yielding fractions Fr3V1-Fr3V13. After combination of fractions as above, Fr3V6 (0.35 g) was purified by CA-TLC (2 mm layer, DCM-EtOAc gradient), yielding fractions Fr3V6C1—Fr3V6C5. Fr3V6C4 (97 mg) was identified as compound **1** by one- (^1^H, ^13^C/APT) and two-dimensional (COSY, HSQC) NMR spectroscopy. Fraction Fr4 (2.8 g) gave crystals on standing in DCM solution at room temperature. The crystals (ca 1.6 g) were filtered off and washed with small amounts of cold DCM, and their purity was tested by TLC. ^1^H and ^13^C/APT NMR spectra were recorded to identify compound **2**. Fraction Fr5 (ca 2.1 g), was dissolved in DCM and applied to a Versapak Si gel column (40 x 150 mm). Elution with a DCM—EtOAc gradient gave nine major fractions, Fr5V1-Fr5V9, which were characterized by ^1^H NMR. Fr5V2 (1.2 g) was further purified by repeated Versapak chromatography to obtain compound **3** (0.50 g). The same compound was also the main component of Fr5V3 (0.45 g). ^1^H and ^13^C/APT NMR spectroscopy was employed to characterize compound **3**. Fraction Fr7 (3.3 g) was rechromatographed over Si gel as above. Fr7V6 (1.7 g) was identified by ^1^H and ^13^C/APT NMR spectroscopy as compound **4**. ^1^H NMR spectral data for the pure substances are shown in Table 1. Spectra of the crude extract have been published previously ([16], suppl.).

**Table 1:** ^1^H NMR data for caryophyllene oxide (1), dihydronitidine (2), sesamin (3) and pellitorine (4) δ values are in ppm, J values in Hz. CDCl_3_ was used as solvent

1	2	3	4
4.97 (1H, d, J 1.3)	7.68 (1H, d, J 8.6)	6.78–6.85 (6H, m)	7.18 (1H, dd, J 10.0, 15.0)
4.86 (1H, d, J 1.3)	7.66 (1H, s)	5.95 (4H, s)	6.10 (1H, m)
2.87 (1H, dd, J 10.7, 4.1)	7.48 (1H, d, J 8.6)	4.72 (2H, d, J 4.4)	6.09 (1H, m)
2.61 (1H, m)	7.31 (1H, s)	4.24 (2H, m)	5.84 (1H, d, J 15.1)
0.85–2.40 (several m)	7.11 (1H, s)	3.88 (2H, dd, J 3.6, 9.3)	3.15 (2H, t, J 6.4)
1.20 (3H, s)	6.79 (1H, s)	3.05 (2H, m)	2.14 (2H, q, J 6.8)
1.01 (3H, s)	6.04 (2H, s)		1.79 (1H, m)
0.99 (3H, s)	4.13 (2H, s)		1.26–1.44 (6H, m)
	3.99 (3H, s)		0.91 (6H, d, J 6.7)
	3.95 (3H, s)		0.89 (3H, t, J 7.0)
	2.60 (3H, s)		

### Adult female mosquitoes bioassays

A non-resistant strain of *An. gambiae* s.s. from Kisumu, Kenya (KIS) was used. Mosquitoes were reared at 25 ± 5 ^o^C and 80 ± 10 % humidity in the laboratory of IRD, Montpellier. Assay for topical toxicity according to standard WHO protocol [17] was done as previously described [16]. Two replicates of 25 non-blood fed 2–5 day-old female mosquitoes (average weight 1.1 mg), were used in each test. Solutions of test substances in different concentrations in acetone (0.1 μL) were applied to the pronotum of each female mosquito, and the number of dead mosquitoes after 24 h was recorded. Acetone alone was used as negative control and acetone solutions of permethrin as positive control.

### Larvae bioassays

Third instar larvae of the same mosquito strain as above were used in larval bioassays according to WHO guidelines [18]. Test solution in ethanol (1 mL) was added to four replicates of 99 mL osmotic water containing 20 larvae. Larvae were kept at 26–28 °C for 24 h and the number of surviving larvae as defined by WHO was counted. Ethanol alone (1 mL) in water was used as negative control. No positive control was carried out.

### Statistics

Non-linear regression analysis was employed to analyse LD_50_ values and confidence intervals (GraphPad Prism 6.05 software).

## Results

### Chemistry

**Fig. 2:**
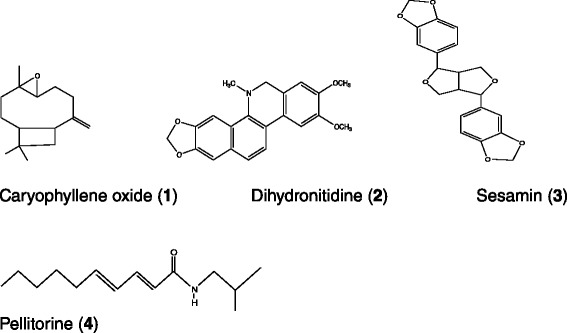
Structures of isolated compounds from *Zanthoxylum heitzii* stem bark

The hexane extract from *Z. heitzii* bark was separated by normal phase chromatography, and after analysis by NMR and TLC twelve fractions were obtained. Based on fraction weights and NMR spectra, Fr3-5 and Fr7 were chosen for further work to identify the major compounds in the extract. This resulted in four major compounds, shown in Fig. 2.

Fraction Fr3V6C4 gave NMR spectra which were consistent with a sesquiterpene structure containing an oxirane ring and a terminal methylene group. From comparison with literature data [19, 20], the substance was identified as caryophyllene oxide (**1**). The crystals from Fr4 gave NMR spectra indicative of an aromatic compound containing one methylenedioxy group, two aromatic methoxyl groups and one N-methyl group. The compound was identified from literature data as the alkaloid dihydronitidine (**2**) [21]. Fractions Fr5V2 and Fr5V3 yielded an aromatic substance with two methylenedioxy groups, as evidenced by NMR. From comparison with literature data [22], the substance was identified as the lignan sesamin (**3**). Fraction Fr7V6 gave a compound containing an isobutyl group, a carbonyl group and two C = C double bonds (NMR). This compound was identified as the amide pellitorine (**4**) from literature data [22]. Data for our spectra are shown in Table 1. Based on weights and estimated purity (from ^1^H NMR), a weight ratio of caryophyllene oxide : dihydronitidine : sesamin : pellitorine in the crude extract of ca 4 : 31 : 26 : 39 was calculated. This is not an analytical result, but only an approximation.

### Insecticidal activity

**Fig. 3:**
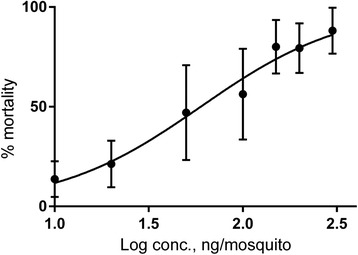
Dose-mortality response of pellitorine towards adult female *An. gambiae* mosquitoes

**Fig. 4:**
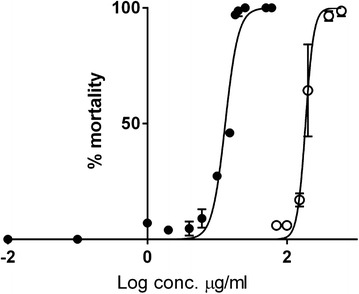
Toxicity of pellitorine (•) and caryophyllene oxide (○) towards *An. gambiae* larvae

Pellitorine showed a toxic effect towards mosquitoes. From nine separate experiments, an LD_50_ value of 50 ng/mg mosquito was found (Table 2), with a nearly linear correlation between log concentration and mortality (Fig. 3). The positive control, permethrin, had an LD_50_ value of 1.9 ng/mg mosquito. For the negative control, acetone, mortality values of 3–5 % were found. The other *Z. heitzii* substances tested were regarded as non-toxic: caryophyllene oxide (6 ± 3 % mortality at 100 ng/mg mosquito, 6 % at 1000 ng/mg mosquito), dihydronitidine (6 ± 3 % mortality at 100 ng/mg mosquito, 23 % mortality at 1000 ng/mg mosquito) and sesamin (9 ± 2 % mortality at 100 ng/mg mosquito, 15 % mortality at 1000 ng/mg mosquito). Due to scarcity of pure substances, the values are averages of two experiments at 1000 ng/mosquito. Since interactions between the different compounds might be present, a mixture of caryophyllene oxide, dihydronitidine, sesamin, and pellitorine was tested. This mixture had approximately the same weight ratio between substances **1**–**4** as in the hexane extract (see above). The mixture showed toxicity towards mosquitoes with an LD_50_ value of 34 ng/mg mosquito. This mixture (100 ng/mg mosquito) produced a mortality of 99 ± 2 % compared to 56 ± 22 % for pellitorine alone (100 ng/mg mosquito). The concentration of pellitorine in the mixture that gives 50 % toxicity is 14 ng/mg mosquito. At this concentration, an expected mortality of ca 18 % is derived from the regression line in Fig. 3; about half of the mortality of the mixture. Pellitorine was the major larvicidal constituent of the extract with an LD_50_ value of 13 μg/ml and 100 % mortality at 25 μg/ml (Fig. 4). Caryophyllene oxide and sesamin showed moderate toxicity (mortality at 150 μg/ml: 22 ± 10 % and 23 ± 3 %, respectively). Dihydronitidine was insoluble in the assay system and was not tested. Ethanol (negative control, 1 % concentration) was non-toxic to mosquito larvae.

**Table 2:** Insecticidal and larvicidal activity against *Anopheles gambiae* of isolated compounds from *Z. heitzii* stem bark

Test compound	Insecticidal activity			Larvicidal activity		
	LD_50_ (ng/mg adult female)	95 % CI	Slope	LD_50_ (μg/ml)	95 % CI	Slope
Caryophyllene oxide (**1**)	>1000			185^e^	175–196	7.4
Dihydronitidine (**2**)	> 1000			nt^a^		
Sesamin (**3**)	> 1000			> 150		
Pellitorine (**4**)	50^c^	43–57	1.1	13^f^	12–14	4.6
Mixture of **1**–**4** ^b^	34^d^	28–38	2.2	nt^a^		
Permethrin (positive control)	1.9^d^	1.7–2.2	1.9	nt^a^		

^a^nt: not tested; ^b^The ratio of compounds **1**–**4** was 4:31:26:39; ^c^9 experiments, each with 2x25 mosquitoes; ^d^4 experiments, each with 2x25 mosquitoes; ^e^5 experiments, 4 with 4x20 larvae, one with 2x20 larvae; ^f^ 4 experiments, 2 with 4x20 larvae, 2 with 2x20 larvae

## Discussion

**Fig. 5:**
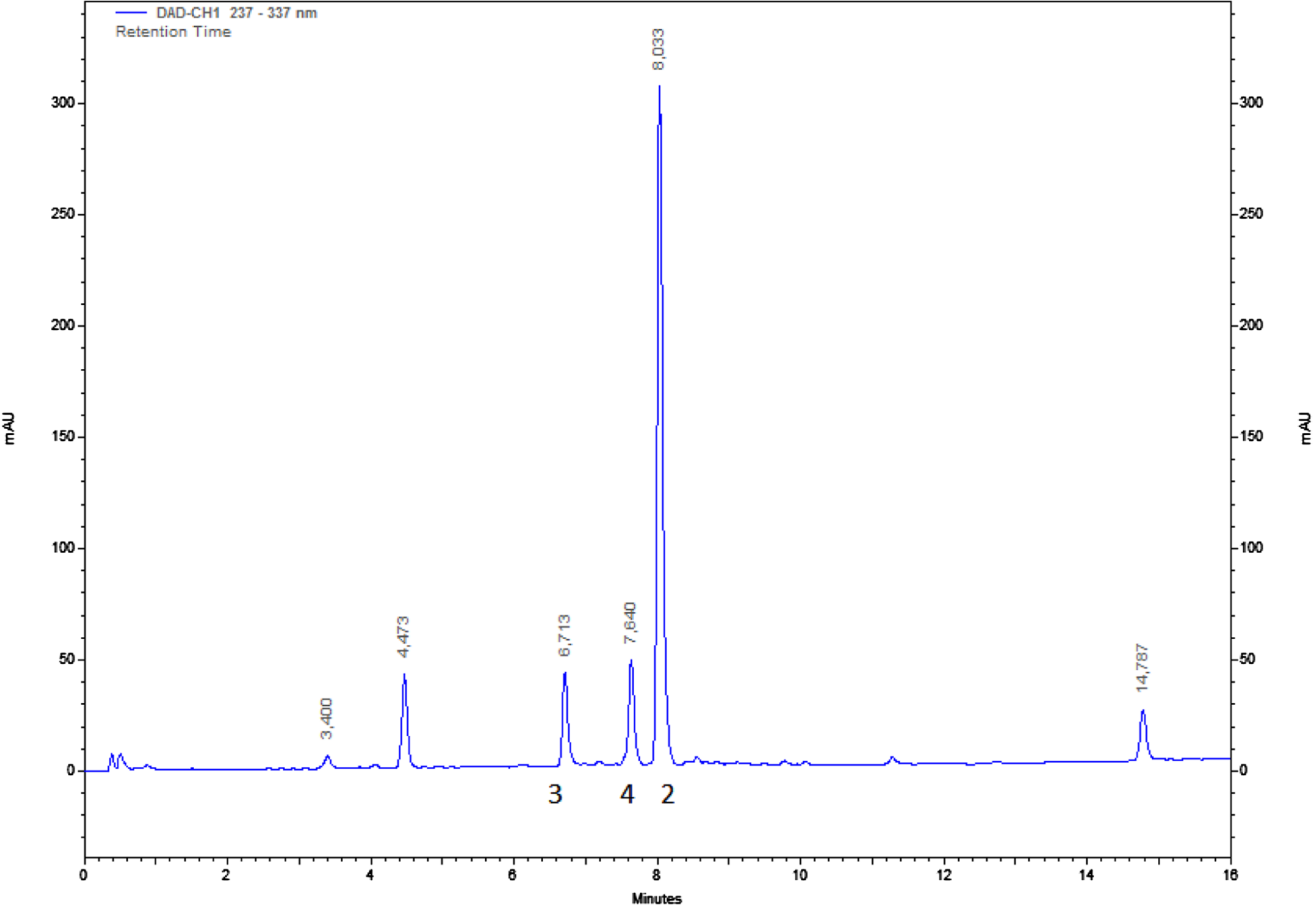
HPLC chromatogram of *Zanthoxylum heitzii* bark hexane extract, UV-detection at 237 nm. **2**: Dihydronitidine; **3**: Sesamin; **4**: Pellitorine

The present work showed that the hexane extract from *Z. heitzii* bark has a relatively simple phytochemical composition (Fig. 5), with pellitorine, dihydronitidine and sesamin as the major compounds (ratio 39:31:26 based on fraction weights and integration of ^1^H NMR spectra of the crude extract). Caryophyllene oxide was found as a minor component. Pellitorine, dihydronitidine and caryophyllene oxide are for the first time (as far as we know) reported from *Z. heitzii*. They have, however, previously been reported from other *Zanthoxylum* species. Sesamin has previously been reported in *Z. heitzii* bark [10, 11]. Caryophyllene oxide is a commonly occurring compound in nature and has previously been found in several *Zanthoxylum* species [23–25], although not in *Z. heitzii*.

Pellitorine was the active component against mosquitoes, although its activity was less than that of the positive control permethrin (LD_50_ 50 vs. 1.9 ng/mg mosquito). This is the first study showing activity of pellitorine towards adult *Anopheles* mosquitoes, although it has been reported to be toxic towards other mosquito species such as *Culex pipiens pallens* L. and *Aedes aegypti* L. [26]. Caryophyllene oxide itself appears not to have been tested previously for toxicity towards adult *An. gambiae* mosquitoes. In our experiments, it was inactive. While caryophyllene oxide often is a constituent of essential oils with effect on mosquitoes [27], it would seem from our results that it is not one of the active components of these oils, although synergistic effects cannot be excluded. Sesamin and dihydronitidine were both inactive against adult *An. gambiae* females. No reports have been found on previous investigations on the toxicity of these compounds against *An. gambiae* adults. However, sesamin is known for synergistic effects together with pyrethroid insecticides which was reported in a study against houseflies in the early 1940s [28], and has since then also been found to exert synergistic activities in other biological systems, e.g. as antihypertensive agent together with vitamin E in rats [29]. The mechanisms behind the synergistic effects are not known in detail. One possibility is that sesamin (a well-known inhibitor of CYP enzymes due partly to its methylene dioxy structure [30, 31]) inhibits cytochrome P450-dependent monooxygenases. The CYP enzymes are present both in insects and mammals and are involved in the metabolism of exogenous compounds, as well as insecticides. Resistance to insecticides may develop due to increased monooxygenase activity which will result in lower insecticide uptake. Therefore, inhibition of CYP450s may be favourable in counteracting insecticide resistance and CYP inhibitors may also increase the uptake and work synergistically with insecticides that are detoxified by the P450s [32]. Terpenoids are also potential synergistic agents, e.g. essential oils which are rich in mono- and sesquiterpenoids have been shown to increase the activity of commercial insecticides [33], although such an effect has apparently not been reported for caryophyllene oxide. It is noteworthy that the mixture of constituents showed higher activity than pellitorine alone, with close to 100 % mortality at 100 ng/mg mosquito. It would seem reasonable that this is due to interaction or synergistic effects between the active constituent pellitorine and the inactive ones, caryophyllene oxide, sesamin and dihydronitidine. This activity is considerably higher than what was found for the crude extract (LD_50_ 102 ± 14 ng/mg mosquito [16]), making it unlikely that other, unidentified components of the extract are important contributors to the total activity of the extract. However, it would be of interest to further study the insecticidal potential of mixtures of these compounds in different ratios to see how variation in the ratio between these components can affect insecticidal activity, and also to study the combination of pellitorine with sesamin as a potential synergistic agent. In view of the increased activity of the mixture of components compared to pellitorine alone, it might also be worthwhile to investigate the possibility of using *Z. heitzii* hexane bark extracts and the active compound pellitorine in combination with other insecticides.

As was the case for adult mosquitoes, pellitorine was the active constituent against mosquito larvae, with an LD_50_ value of 13 μg/ml. Pellitorine has not been reported previously to be toxic towards *An. gambiae* larvae, although the compound is known to be toxic towards larvae of other mosquitoes such as *Culex pipiens pallens* and *Aedes aegypti* [34, 35]. Caryophyllene oxide and sesamin had only low activity on *An. gambiae* larvae, exhibiting a mortality of ca 20 % at 150 μg/ml. While an essential oil with larvicidal properties contains caryophyllene oxide as one of many constituents [36], the putative larvicidal effect of caryophyllene oxide itself has, to our knowledge, not been reported. No previous reports on the toxicity of sesamin towards *An. gambiae* larvae have been found. Dihydronitidine could not be tested in this assay due to low solubility. In view of its lack of activity towards adult mosquitoes and towards brine shrimp larvae (unpublished results), it would seem unlikely that it is a major toxin for *An. gambiae* larvae. No previous reports on the toxicity of dihydronitidine towards *An. gambiae* larvae have been found.

## Conclusions

In summary, the toxic effect of hexane extracts of *Z. heitzii* stem bark towards adult insects of the malaria vector *Anopheles gambiae* [16] appears to be due to the pellitorine content of the extract. An interesting finding is that the activity of pellitorine is enhanced by admixture with other, inactive constituents of the extract. Pellitorine is toxic to *An. gambiae* larvae, as well. This is the first report of pellitorine toxicity towards *Anopheles gambiae* adults and larvae.
